# Genetic Characterization of Toggenburg Orbivirus, a New Bluetongue Virus, from Goats, Switzerland 

**DOI:** 10.3201/eid1412.080818

**Published:** 2008-12

**Authors:** Martin A. Hofmann, Sandra Renzullo, Markus Mader, Valérie Chaignat, Gabriella Worwa, Barbara Thuer

**Affiliations:** Institute of Virology and Immunoprophylaxis, Mittelhaeusern, Switzerland

**Keywords:** Orbivirus, bluetongue virus, goats, molecular epidemiology, genotyping, serotypes, research

## Abstract

A novel bluetongue virus (BTV) termed Toggenburg orbivirus (TOV) was detected in goats from Switzerland by using real-time reverse transcription–PCR. cDNA corresponding to the complete sequence of 7 of 10 double-stranded RNA segments of the viral genome was amplified by PCR and cloned into a plasmid vector. Five clones for each genome segment were sequenced to determine a consensus sequence. BLAST analysis and dendrogram construction showed that TOV is closely related to BTV, although some genome segments are distinct from the 24 known BTV serotypes. Maximal sequence identity to any BTV ranged from 63% (segment 2) to 79% (segments 7 and 10). Because the gene encoding outer capsid protein 2 (VP2), which determines the serotype of BTV, is placed within the BTV serogroup, we propose that TOV represents an unknown 25th serotype of BTV.

## Abstract 2

Nucleotide sequences analysis indicates that this virus is a new serotype of bluetongue virus.

Bluetongue is a vectorborne disease affecting all ruminant species but causing clinical disease mostly in sheep; cattle and goats are usually considered asymptomatic reservoir hosts (*1*). The disease occurs worldwide and is caused by bluetongue virus (BTV), which belongs to the genus *Orbivirus* within the family *Reoviridae*. Other species of the orbiviruses are African horse sickness virus, epizootic hemorrhagic disease virus (EHDV), and some lesser-known viruses such as Peruvian horse sickness virus, Chuzan virus, Saint Croix River virus, and Yunnan orbivirus (*2*). The virus has a segmented double-stranded RNA genome consisting of 10 segments that code for 11 viral proteins. BTV is transmitted between its ruminant host mainly by blood-feeding midges of the *Culicoides* spp. Twenty-four serotypes of BTV can be distinguished on the basis of the antigenic profile of its major outer capsid protein VP2 (*3*).

In Europe, several serotypes of BTV were detected sporadically in the 1990s (*4*). From 1998 through 2006, a total of 5 serotypes (1, 2, 4, 9, and 16) became established in southern Europe (*5*), but they were kept confined to this area, most likely because their main insect vector, *C*. *imicola*, has never been detected north of the Alps in Europe (*6*). A BTV-monitoring program in cattle herds has been in effect in Switzerland since 2003 in Ticino Canton, south of the Alps (*7*–*9*). However, no BTV has been detected until now in this region of the country. Furthermore, northern Europe had never been affected by bluetongue before 2006, although BTV can also be transmitted by other *Culicoides* species, such as *C. obsoletus* and *C. pulicaris* (*6*), which are native to this area.

In 2006, BTV serotype 8 (BTV-8) of likely African origin was introduced into Belgium by an as yet unknown mechanism (*8*). This virus spread throughout many northern European countries. By mid-2008, BTV-8 had caused numerous clinical bluetongue cases in sheep and cattle in the Netherlands, Belgium, Germany, Luxembourg, France, Denmark, Czech Republic, United Kingdom, Italy, Spain, and Switzerland (*5*).

The first clinical case of bluetongue caused by BTV-8 in Switzerland was detected in October 2007 on a dairy cattle farm (*10*). Because Switzerland was already included in a surveillance zone and, after detection of the first BTV-8 infection, a protection zone, all animals were tested serologically and virologically before trade. Four additional cases of BTV-8 infection were detected from October through November 2007 (*10*,*11*).

Early in 2008, several BTV-positive, clinically healthy animals were detected in a goat flock in northeastern Switzerland (V. Chaignat et al., unpub. data) by BTV-specific antibody ELISAs and viral RNA detection, by using a BTV-specific real-time reverse transcription–PCR (rRT-PCR) (*10*,*12*). However, levels of antibody and viral RNA were unexpectedly low. Furthermore, presence of BTV RNA could not be confirmed by use of other BTV-specific rRT-PCR protocols (*13*,*14*), and amplification curves obtained in the screening rRT-PCR suggested that the target sequence on RNA segment 10 of this virus might be different from all known BTV strains (data not shown). For this reason, we determined the nucleotide sequence of the rRT-PCR amplification product.

Experimental infection of goats and sheep by subcutaneous and intravenous injection of blood from virus-positive animals from the herd in which the infection was first discovered demonstrated that the virus is transmissible to and replicates in goats, albeit without causing bluetongue-specific clinical symptoms. In contrast, the virus could only be sporadically detected in inoculated sheep (V. Chaignat et al., unpub. data). On the basis of the sequence of the rRT-PCR product, this newly detected virus, termed Toggenburg orbivirus (TOV), could represent an unknown orbivirus of low pathogenicity, or a new serotype of BTV. Thus, we performed a detailed genetic characterization of the TOV genome.

The complete coding sequence for the 7 RNA segments (2, 5, 6, 7, 8, 9, and 10) was determined. Segment 2 was chosen because it encodes the viral structural protein VP2 that contains the serotype-specific determinants, whereas the genome segments 5 through 10 are short enough to enable sequencing of the cloned cDNA without the need for internal sequencing primers. We present evidence that TOV is genetically related to BTV but cannot be assigned to any of the 24 known serotypes; instead, it likely represents a 25th BTV serotype.

## Materials and Methods

### Source of Viral RNA and Extraction of RNA

BTV-like orbivirus RNA and antibodies were detected in several adult and newborn goats in a herd in the northeastern part of Switzerland (St. Gallen Canton) (V. Chaignat et al., unpub. data). We used erythrocytes from blood collected into tubes containing EDTA from 1 adult and 1 newborn goat, which had cycle threshold (C_T_) values of 22 and 32, respectively, in the rRT-PCR (*10*). Total RNA was extracted from 250 μL of erythrocyte suspension by using Trizol (Invitrogen, Basel, Switzerland) according to the manufacturer’s instructions. Precipitated purified RNA was dissolved in 20 μL of RNase-free water.

### RT-PCR for Full-Length cDNA Amplification, Cloning, and Sequencing

Primers corresponding to inter-BTV serotype-conserved 5′ and 3′ terminal sequences were designed for each of the viral RNA segments to be analyzed (Table 1). Before RT-PCR, 8 μL of extracted viral RNA was mixed with 50 pM of 2 RNA segment-specific forward and reverse primers and heat-denatured for 5 min at 95°C. Reverse transcription of both RNA strands into double-stranded cDNA was performed by using SuperScript III reverse transcriptase (Invitrogen) in a final volume of 25 μL. A total of 8 μL of cDNA was then added to a PCR mixture containing Platinum Taq High Fidelity Polymerase (Invitrogen) and amplified by 40 cycles at 95°C for 30 s, 50°C for 1 min, and 72°C for 1 min (for segments 5–10) or 72°C for 8 min (for segment 2).

**Table 1:** Terminal primers used to amplify complete Toggenburg orbivirus RNA segments

RNA segment	Name*	Sequence (5′ → 3′)
	Forward primer	
2	BTV4&10_S2_-21_F	GGGTTAAAAGAGTGTTCYAC
5	BTV_S5_F	GTTAAAAAAGTTCTCTAGTTGGCA
6	BTV4&10&11_S6_-30_F	GTTAAAAAGTRTTCTCCTACTC
7	BTV_S7_-17_F	GTTAAAAATCTATAGAGATGGAC
8	BTV_S8_-19_F	GTTAAAAAAWCCTTGAGTCATG
9	BTV_S9_-15_F	GTTAAAAAATCGCATATGTCAG
10	BTV_S10_F	GTTAAAAAGTGTCGCTGCCAT
	Reverse primer	
2	BTV4&20_S2_R	GTAAGTGTAAGAAGGCCACAG
5	BTV_S5_R	GTAAGTTGAAAAGTTCTAGTAGAG
6	BTV4&10_S6_1618_R	GTAAGTGTAATCTTCTCCCTC
7	BTV_S7_1142_R	GTAAGTGTAATCTAAGAGACGT
8	BTV_S8_1106_R	GTAAGTGTAAAATCCCCCCC
9	BTV_S9_1038_R	GTAAGTRTGAAATCGCCCTAC
10	BTV_S10_R	ACCTYGGGGCGCCACTC

*BTV, bluetongue virus.

PCR-amplified cDNA of the expected length was purified by agarose gel electrophoresis and ligated into plasmid vector pCR4-TOPO (Invitrogen). Clones of transformed *Escherichia coli* harboring the TOV insert-containing vector were identified by PCR preps using insert-spanning M13 primers. Miniprep DNA of >5 cDNA clones from each viral genome segment was sequenced by cycle sequencing using IRD800 and IRD700 infrared dye–labeled M13 (Eurofins MWG Operon, Ebersberg, Germany) and TOV segment 2–specific internal primers. Sequencing reactions were subjected to electrophoresis in a 4300L DNA sequencer (LI-COR, Lincoln, NE, USA) and analyzed by using e-Seq V3.0 and AlignIR V2.0 software (LI-COR). The coding region of each TOV genome segment analyzed in this study was compared with published orbivirus sequences by using online BLAST analysis (http://blast.ncbi.nlm.nih.gov/blast.cgi). For phylogenetic analysis, TOV-specific sequences were aligned to a selection of available corresponding sequences from GenBank that represented all orbivirus species by using MEGA version 4.0 software (*15*) with default parameters. The open reading frame (ORF) sequences of the 7 analyzed genome segments of TOV were submitted to GenBank under accession nos. EU839840 (S2), EU839841 (S5), EU839842 (S6), EU839843 (S7), EU839844 (S8), EU839845 (S9), and EU839846 (S10).

## Results

**Figure 1:**
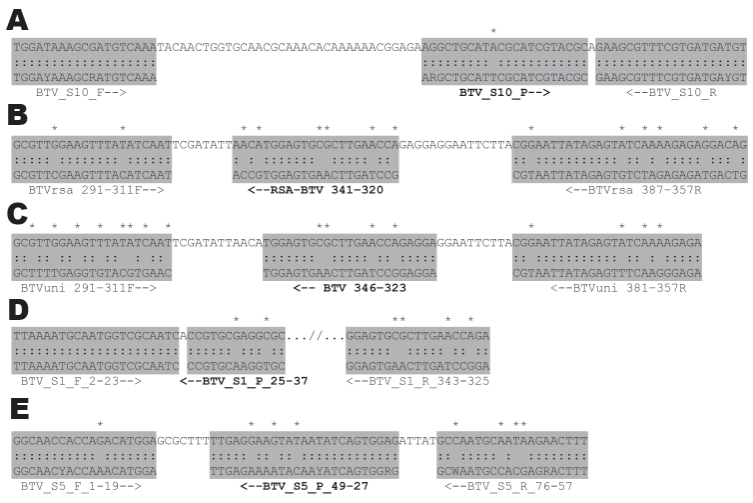
Nucleotide sequence alignment of target regions of published bluetongue virus (BTV) real-time reverse transcription–PCR primers and probes (in **boldface**), which were used for detection of Toggenburg orbivirus (TOV). A) Segment 10 (*10*); B) segment 1, eastern serotype specific (*13*); C) segment 1, western serotype specific (*13*); D) segment 1 (*14*); E), segment 5 (*14*). Shaded areas indicate primer and probe sequences (in sense orientation), colons indicate sequence identity, arrows indicate orientations of probes and primers, and asterisks indicate mismatches between primers/probes and TOV genome sequence.

TOV was initially detected by using an RNA segment 10–specific rRT-PCR (*10*). Nucleotide sequencing of this rRT-PCR product indicated that TOV is an orbivirus closely related to, but not identical with, any known BTV serotype. The analyzed sequence showed a 1-base mismatch in the probe region of the rRT-PCR target sequence (Figure 1, panel **A**), which could explain the unusual amplification curves (low delta Rn value, the magnitude of the signal generated by the given set of PCR conditions). Various additional rRT-PCR protocols all yielded negative results (data not shown). When the primer and probe sequences of these published rRT-PCR protocols for segment 1 (*13*,*14*) or segment 5 (*14*), respectively, were aligned to the corresponding TOV sequences determined in this study, numerous mismatches were found (Figure 1). These mismatches were the likely reason for the failure of these assays to detect TOV. Because sequence amplified by the rRT-PCR (Figure 1, panel **A**) was most closely related to several BTV-4 and BTV-10 serotypes, RNA segment–specific primers binding to the conserved 3′ and 5′ untranslated regions were designed on the basis of these serotypes whenever no consensus sequence matching with all published sequences (representing all 24 serotypes) could be found, i.e., for segments 2 and 6.

Although the amount of viral RNA in blood from infected but clinically healthy goats was low (C_T_ values >30 in the rRT-PCR) we could amplify the full-length cDNA sequence for all 7 RNA segments analyzed, including the 2.9-kb fragment of segment 2. PCR products all showed the expected length as predicted from the corresponding BTV genome segment. After cloning into the pCR4-TOPO vector, both DNA strands of 5 clones were sequenced for each genome segment. A consensus sequence could be unambiguously determined because the number of point mutations in the clones was low, and never more than 1 of the 5 clones showed a difference from the consensus sequence (data not shown).

BLAST analysis of the 7 TOV genome segments showed in all cases the highest sequence similarity to BTV, although the search algorithm had to be changed from megablast (highly similar sequences) to blastn (somewhat similar sequences) to detect any sequence homology. However, results varied widely in terms of percentage of identity to their closest BTV sequence and to BTV serotype showing the highest similarity (Table 2). No BTV strain was distinctly more closely related to TOV than all other known sequences, although for several genome segments BTV serotype 4 isolates were found to be the closest relatives to TOV. When identity levels between analyzed TOV genome segments to their genetically closest BTV strain were compared with respective values of the 2 most distantly related BTV serotypes, TOV sequences were in some cases (segments 5, 8, and 10) clearly more different than the BTV sequences among themselves. However, for the remaining segments, TOV sequences were less different from their closest BTV sequence than the 2 most distantly related BTV serotypes (segments 2, 6, 7, and 9).

**Table 2:** Sequence comparisons of Toggenburg orbivirus genes by BLAST analyses*

RNA segment	BLAST best hit					
	Serotype	Isolate	Accession no.	No. identities/total	Identity level, %	Query coverage, %
2	4	Turkey 1999	DQ825670	1,855/2,919	63	100
5	16	TUR2000/10	AM773696	1,242/1,662	75	100
6	4	ARG2002/01	AJ586682	1,163/1,585	73	100
7	23	Dehradoon	AJ277802	832/1,050	79	100
8	4	Corsica	AY857499	778/1,072	72	100
9	10	10O90H	BVU55782	742/1,005	73	100
10	8	8438/vaccine	AF512919	550/694	79	100

*Only coding regions of Toggenburg orbivirus genes (including stop codons) were used (http://blast.ncbi.nlm.nih.gov/blast.cgi).

**Figure 2:**
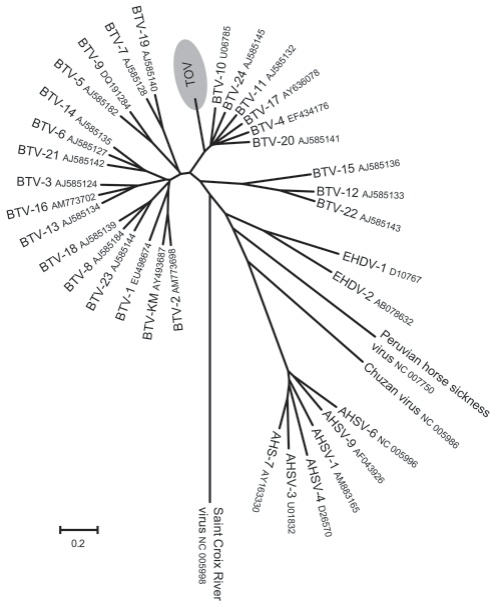
Phylogenetic analysis of Toggenburg orbivirus (TOV) (shaded region) genome segment 2 by ClustalW alignment (*16*) and subsequent neighbor-joining tree construction by MEGA version 4 software (*15*). GenBank accession numbers are indicated for all orbivirus sequences used to construct dendrogram. BTV, bluetongue virus; EHDV, epizootic hemorrhagic disease virus; AHSV, African horse sickness virus. Scale bar indicates number of nucleotide substitutions per site.

**Figure 3:**
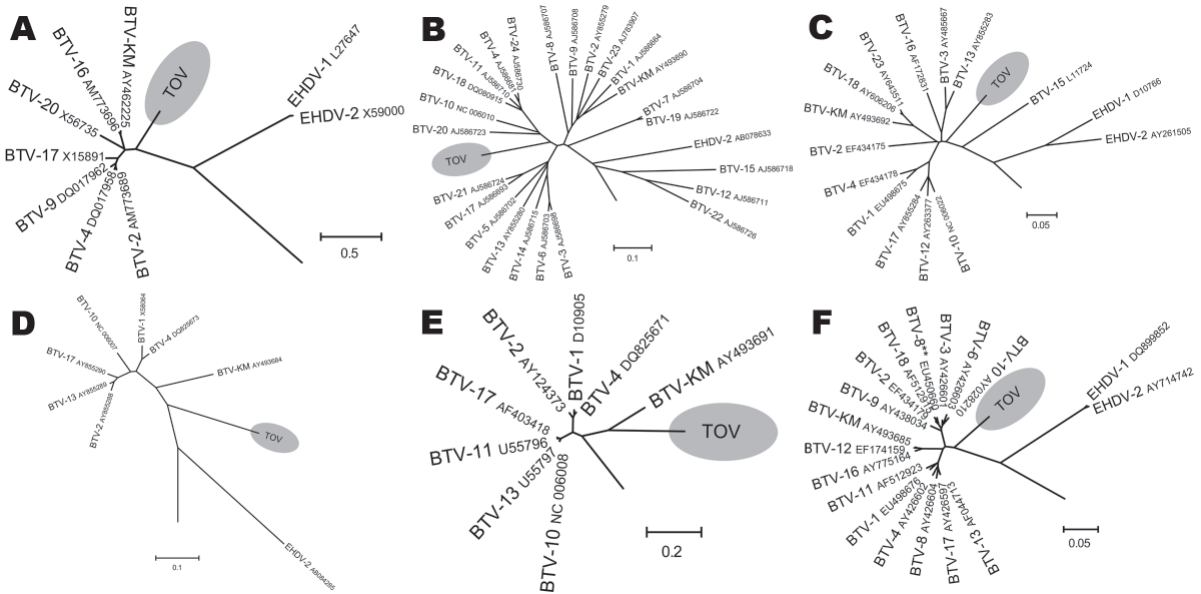
Phylogenetic analysis of Toggenburg orbivirus (TOV) (shaded regions) genome segments by ClustalW alignment (*16*) and subsequent neighbor-joining tree construction by MEGA version 4 software (*15*). GenBank accession numbers are indicated for all orbivirus sequences used to construct dendrograms. A) Segment 5; B) segment 6; C) segment 7; D) segment 8; E) segment 9; F) segment 10. BTV, bluetongue virus; EHDV, epizootic hemorrhagic disease virus. Segments show only relevant parts of dendrograms. **Segment 10 sequence of BTV-8 currently circulating in northern Europe. Scale bars indicate number of nucleotide substitutions per site.

Results of phylogenetic analysis using ClustalW alignment (*16*) and neighbor-joining tree construction confirmed the results of BLAST analyses by locating some TOV genome segments within the BTV serogroup (segments 2, 6, 7, and 9). In contrast, for segments 5, 8, and 10, TOV sequences were outside the BTV subtree (Figures 2, 3). Furthermore, the S2 gene could not be assigned to any of the 9 proposed nucleotypes (*17*) (data not shown). Despite the different placement of the 7 TOV genome segments, they were all more closely related to BTV than to any of the other known orbivirus species, in particular to EHDV, which is the closest relative to BTV (Figures 2, 3).

## Discussion

In the context of trade investigations implemented in Switzerland shortly after the first BTV-8 outbreak was detected (*11*), BTV-specific RNA was detected in clinically healthy goats in St. Gallen Canton in northeastern Switzerland by segment 10–specific rRT-PCR. However, none of the additional rRT-PCR protocols for other BTV genome segments, which were performed for confirmatory purposes, yielded positive results. Furthermore, the short (58-nt) segment 10–specific sequence amplified by the initial rRT-PCR was similar but not identical to any known BTV. Therefore, to characterize TOV as a potentially novel BTV, we performed a detailed genome analysis by cloning and sequencing the entire coding region of most viral genome segments.

cDNA of smaller genome segments that encode the structural proteins VP5 (segment 6), VP6 (segment 9), VP7 (segment 7), and nonstructural (NS) proteins NS1 (segment 5), NS2 (segment 8), and NS3/NS3A (segment 10) could be amplified and sequenced without the need for internal, TOV-specific primers. The 2.9-kb region spanning segment 2 was also included; however, a primer-walking approach had to be used for design of TOV-specific sequencing primers (data not shown). VP2, which is encoded by segment 2, is exposed at the virion surface, together with VP5 (*3*), and carries the epitopes inducing serotype-specific neutralizing antibodies (*18*). Thus, segment 2 was expected to contain the genetic information enabling assignment of TOV to 1 of the 24 BTV serotypes.

Although initial attempts to isolate TOV in several mammalian cell lines failed, there is preliminary evidence that the virus replicates in the highly BTV-susceptible KC insect cell line derived from *Culicoides* spp. (*19*). However, to avoid introduction of mutations in the viral genome caused by cell culture adaptation, we used blood as the source for cloning and nucleotide sequencing of the TOV genome.

For accurate identification of a consensus sequence for each cloned TOV genome segment, 5 clones were analyzed for each segment. When sequences of individual clones were compared, mismatches were found in <1% of the nucleotide positions (data not shown), and always in only 1 of the 5 clones. If one takes into account the error rate of the reverse transcriptase and Taq DNA polymerase used to transform the viral RNA into PCR-amplified cDNA, this finding supports reported genetic stability of the double-stranded RNA genome of orbiviruses (*20*).

BLAST analysis of the ORF sequence of the 7 TOV genome segments studied suggested that TOV most likely belongs to the BTV serogroup because although the degree of nucleotide sequence identity between TOV and the closest BTV relative was low, each of the TOV genome segments was closely related to BTV and not to another orbivirus. The BLAST results were confirmed by individual phylogenetic analysis of the 7 TOV genome segments. Depending on the segment, TOV was placed in a different neighborhood among BTV and EHDV (Figures 2, 3). Although segments 2, 6, 7, and 9 encoding viral structural proteins were placed within the BTV subtree in the dendrograms, albeit being distinct from all 24 BTV serotypes, the remaining segments 5, 8, and 10 coding for NS proteins were located outside the branching area of the BTV subtree toward the EHDV subtree. These findings suggest that TOV might have diverged from BTV and evolved independently or could represent a reassortant between BTV and an unknown orbivirus closely related to EHDV. This assumption is supported by results of BLAST analysis that showed a greater evolutionary distance between TOV and BTV than among the various BTV serotypes (data not shown), which is evident in the case of the segment 10 encoding NS3/NS3A. It will be useful to determine whether TOV genome segments 1, 3, and 4, which encode viral structural proteins, are also more closely related to BTV than the NS genes.

Definitive characterization of TOV as a new BTV serotype will require a comprehensive serologic typing with BTV serotype–specific antisera and TOV-specific antisera. It is likely that TOV represents a new BTV serotype because agreement of segment 2–based genotypic and serology-based phenotypic serotype differentiation, involving a large collection of BTV strains representing all 24 known serotypes, has been reported (*17*).

We propose that TOV more likely represents an unknown 25th serotype of BTV rather than a new orbivirus species, on the basis of grouping of structural protein genes within BTV and because sera from goats from which the virus was isolated showed reactivity in several BTV-specific antibody ELISAs. These tests did not show reactivity with antibodies against EHDV, which represents the closest relative to BTV among the orbiviruses (data not shown).

TOV did not cause bluetongue-specific clinical signs in experimentally infected goats (V. Chaignat et al., unpub. data). In addition, virus load was low. These findings are consistent with the fact that goats generally show no clinical disease after BTV infection but can serve as reservoir hosts (*1*). However, TOV was only sporadically detected by rRT-PCR in inoculated sheep, and these animals did not show any pronounced clinical signs of bluetongue. If TOV also shows an apathogenic phenotype in other ruminants, this finding would further support the possibility that TOV may have evolved differently than classic BTV strains. Identification of insect vector(s) for TOV would be useful because these vector(s) might influence transmission and phenotypic properties of TOV.

An apathogenic BTV termed the KM strain has been reported in goats from Taiwan (*21*). This virus was characterized as a typical BTV and was added to BTV reference sequences for the present phylogenetic analysis (Figures 2, 3). On the basis of its high segment 2 sequence similarity with BTV-2 (Figure 2), it is likely that KM belongs to this BTV serotype. Comparison of TOV with KM shows that TOV is less related to classic BTV than KM, which rules out TOV and the KM strain having a common ancestor, although the 2 viruses share a high sequence homology in their segment 9 ORF (Figure 3). Therefore, TOV might represent a reassortant BTV that acquired its segment 9 from a BTV closely related to KM.

To determine the genetic relationship of TOV with the BTV-8 strain currently circulating in northern Europe, the segment 10 coding region of the Swiss BTV-8 isolates from 2 of the late 2007 outbreaks was sequenced and submitted to GenBank (accession nos. EU450660 and EU450661). This BTV-8 was different from a BTV-8 reference strain obtained from the Office International des Epizooties Reference Laboratory for bluetongue (Institute of Animal Health, Pirbright, UK) (accession no. EU450663). Although TOV was initially detected in Switzerland at the same time as the first BTV-8 infections, these 2 BTV strains are genetically diverse, as shown in the dendrogram of segment 10 (Figure 3). None of the 7 genome segments analyzed or any of the 3 remaining genome segments (1, 3, and 4) (data not shown), showed sequence homologies >79% with any other BTV, and none of the BLAST analyses showed a maximal sequence identity level with the corresponding genome segment of the BTV-8 currently circulating in Europe (*22*). These findings indicate that TOV is not a reassortant between BTV-8 and another BTV.

Distinct sequence differences on RNA segments 1 and 5 may also explain why TOV was not detectable by rRT-PCR protocols designed to detect a wide range of BTV serotypes (Figure 1). Only the segment 10–specific rRT-PCR showed a positive result, whereas all segment 5– and segment 1–specific assays did not detect TOV. These findings demonstrate the importance of selecting an appropriate rRT-PCR protocol for detection of TOV and other unusual BTV-like viruses. The fact that most diagnostic laboratories use segment 1– or segment 5–specific rRT-PCRs for routine detection of BTV might also explain why TOV has never been detected in other regions.

TOV and other BTV-25 strains, as potential apathogenic viruses, will have major implications in control of bluetongue. A similar situation is found in the United States, where several BTV serotypes circulate without causing any clinical signs in domestic ruminants; nevertheless, national and international animal trade is heavily affected (*23*).

Additional animal experiments in various ruminant species and adaptation of TOV to cell culture for extended phenotypic characterization (e.g., replication kinetics) are currently under way. TOV-specific rRT-PCR protocols will be developed to determine TOV prevalence and to clarify its role as an animal pathogen. Furthermore, TOV-specific serologic tools that use immunologically dominant epitopes of VP2 and VP5 as recombinant ELISA antigens for detection of antibodies to TOV should be developed. These tools would facilitate epidemiologic studies to determine actual and retrospective seroprevalences of TOV in goats and other domestic and wild ruminants in Switzerland and throughout the world, in particular in countries that have long claimed to be free of BTV infections. Furthermore, TOV-specific diagnostic tools will enable identification of natural reservoir(s) and address how and by which insect vectors this orbivirus is transmitted.
